# Global myocardial work in coronary artery disease patients without regional wall motion abnormality: Correlation with Gensini‐score

**DOI:** 10.1002/clc.24193

**Published:** 2023-11-28

**Authors:** Miao Li, Yuhao Wang, Lin Li, Wenfang Wu, Rong Qian, Pingyang Zhang

**Affiliations:** ^1^ Department of Cardiovascular Ultrasound, Nanjing First Hospital Nanjing Medical University Nanjing China; ^2^ Department of Ultrasound, No. 905 Hospital of People's Liberation Army Navy Naval Medical University Shanghai China

**Keywords:** coronary artery disease, Gensini‐score, global longitudinal strain, myocardial work, pressure‐strain loops

## Abstract

**Background:**

Early detection of coronary atherosclerotic diseases (CAD) without regional wall motion abnormality (RWMA) is important for improving the outcome of cardiovascular events. Global myocardial work (GMW), including global myocardial work index (GWI), global constructive work (GCW), global wasted work (GWW), and global myocardial work efficiency (GWE), offer comprehensive quantitative assessment of myocardial function in CAD.

**Hypothesis:**

We hypothesized that GMW could provide incremental value in detecting CAD without RWMA.

**Methods:**

One hundred and twenty‐four patients referred for coronary angiography (CAG) without resting RWMA were enrolled in this study. Global longitudinal strain (GLS), GWI, GCW, GWW, GWE were quantified. The severity of coronary lesions was evaluated by Gensini score (GS) based on CAG. We further divided CAG‐confirmed CAD patients into three subgroups according to the tertiles of GS: low 0 < GS ≤ 17, mid 17 < GS ≤ 38, and high GS > 38.

**Results:**

Compared with control, CAD patients showed decreased GLS, GWE, GWI, GCW but an increased GWW. Compared to low‐GS group, GWW was increased in the mid‐GS group. GLS, GWE, GWI and GCW were decreased in the high‐GS group while GWW was increased. Receiver operator characteristic curve analysis demonstrated that GWE was the most powerful predictor of high‐GS and was superior to GLS. GWE under 92.0% had the optimal sensitivity and specificity for identifying high‐GS.

**Conclusion:**

The proposed GWE, which outperformed the conventional GLS, could be considered as a potential predictive indicator to help to detect severe coronary disease in non‐RWMA CAD patients.

## Graphical abstract

In this retrospective study of 124 patients, we investigated the global myocardial work (GMW) in coronary atherosclerotic diseases (CAD) patients without regional wall motion abnormality (RWMA) and explored the relationship between GMW and severity of CAD using Gensini score (GS). Our results show that global myocardial work efficiency (GWE) was the most powerful predictor of high‐GS and was superior to conventional global longitudinal strain. GWE could be considered as a potential predictive indicator to help to detect severe coronary disease in non‐RWMA CAD patients.

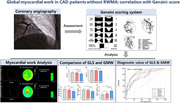

## 1 INTRODUCTION

Coronary atherosclerotic diseases (CAD) remain the leading cause of mortality and morbidity in patients with cardiovascular diseases.^1^ Early detection of coronary artery lesions has a paramount impact on clinical decision‐making and prognosis improvement. Currently, coronary angiography (CAG) is considered a standard tool for the diagnosis of CAD and the assessment of the severity of coronary lesion. The Gensini score (GS) based on CAG findings has become an effective parameter in predicting survival outcomes and cardiovascular events.^2^, ^3^ However, as an invasive examination method, CAG carries certain risks, particularly for patients with poor general conditions. Therefore, it is of utmost importance to seek a noninvasive method to detect CAD and evaluate the degree of coronary artery stenosis.

Transthoracic echocardiography (TTE) is a widely accepted noninvasive technique in diagnosing CAD based on detecting decreased left ventricular ejection fraction (LVEF) and regional wall motion abnormality (RWMA). However, during the early stage of coronary atherosclerosis, unless there is an evident ischemia or infarction, RWMAs are usually invisible and the LV functional parameters tend to be preserved. Previous studies reported that myocardial strain analysis has the potential to be an efficient tool in the detection of myocardial ischemia, microvascular dysfunction, and diagnostics of coronary artery stenosis in patients with preserved LVEF and normal resting LV wall motion.^4^, ^5^, ^6^, ^7^ Speckle‐tracking echocardiography (STE) focusing on myocardial strain analysis provides a new perspective to quantify myocardial function beyond conventional assessment of RWMAs. However, it should be noted that the diagnostic accuracy of STE in evaluating myocardial function is influenced by load dependence, which is a significant factor.^8^

Myocardial work (MW) analysis is a novel method that accounts for myocardial strain as well as afterload offering comprehensive quantitative assessment of myocardial function.^8^, ^9^, ^10^, ^11^, ^12^, ^13^ A recent study by Boe et al. revealed that in patients with non‐ST‐segment elevation acute coronary syndrome (NSTE‐ACS), regional MW analysis can be used to identify the presence of acute coronary occlusion (ACO).^9^

The objective of this study is to explore the feasibility of MW analysis in CAD detection, and identify the correlation between MW and coronary lesion severity determined from the GS.

## 2 MATERIALS AND METHODS

### 2.1 Clinical enrollment

**Figure 1:**
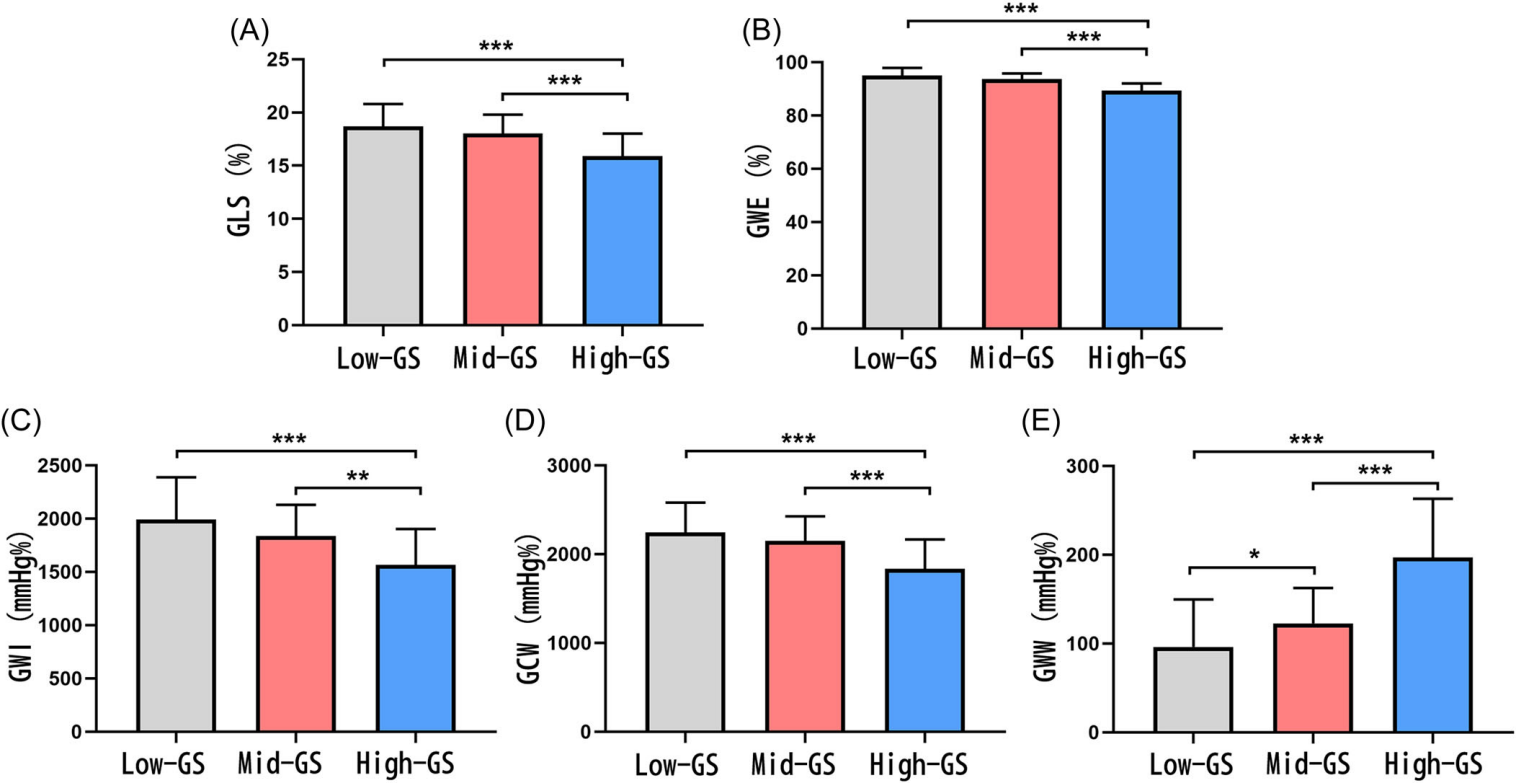
Comparison of global longitudinal strain (GLS) and global myocardial work (GMW) parameters among three Gensini score (GS) groups. Comparison of GLS (A) and global myocardial work efficiency (GWE) (B), global myocardial work index (GWI) (C), global constructive work (GCW) (D), global wasted work (GWW) (E) among three GS groups. *p < .05, **p < .01, ***p < .001.

This study was a retrospective study involving a cohort of 155 patients suspected of having coronary artery disease (CAD) and referred for CAG at Nanjing First Hospital between January 2022 and August 2022. The inclusion of patients in this study was based on their symptoms (such as a history of chest pain, stable angina symptoms, or exertional dyspnea) and/or laboratory findings (positive or suspected results on exercise stress testing or computed tomographic CAG). Notably, only patients in a stable condition were included, while those with suspected acute coronary syndrome, a history of myocardial infarction or revascularization, were excluded. TTE was performed within 12 h before CAG for all patients. Exclusion criteria comprised evident RWMA on TTE and/or LVEF below 55% (*n* = 10), aortic stenosis (*n* = 3), left ventricular outflow tract (LVOT) obstruction (*n* = 2), severe arrhythmia, valvular diseases, cardiomyopathy, poor echocardiographic image quality, and other significant systemic diseases (see Supporting Information S1: Figure 1 for detailed information). Ultimately, 124 patients who met the inclusion criteria were included in the analysis. All patients enrolled in this study remained stable during the imaging examinations.

The present study was conducted in accordance with the principles outlined by the Declaration of Helsinki and received approval from the Ethics Committees of Nanjing First Hospital. Informed consent was obtained from all individual participants included in the study.

### 2.2 Routine TTE

All conventional two‐dimensional and doppler echocardiographic measurements were performed following the guidelines outlined of the American Society of Echocardiography.^14^, ^15^ During the TTE examination, continuous electrocardiograph (ECG) monitoring was conducted for all participants. TTE was performed within 12 h before CAG using a Vivid E95 ultrasound system (GE Healthcare) equipped with an M5S transducer (3.5 MHz). Stroke volume (SV) and LVEF (%) were calculated using the modified bi‐plane Simpson method. peak E/A ratio and peak E/e ratio of the mitral valve (MV) were obtained by spectral Doppler and tissue Doppler imaging.

### 2.3 Two‐dimensional STE

For offline analysis, standard two‐dimensional images were acquired in apical long‐axis, apical two‐chamber, and apical four‐chamber views, each consisting of three cardiac cycles. Echocardiographic images were interpreted by two experienced sonographers blind to each other's findings and clinical information. To ensure successful implementation of strain imaging, frame rates were set at 50 and 80 frames per second, with a mean of 55.6 ± 2.9 frames per second. Global longitudinal strain (GLS) was quantified using semi‐automated functional imaging using EchoPAC software (version 203, GE Vingmed Ultrasound). Once GLS was determined, the timing of aortic and mitral valve closure and opening were confirmed by aligning the event time visually in the apical long‐axis view. An automatic algorithm was used to trace and track the left ventricular (LV) myocardium, with manual adjustment of the endocardium boundary if necessary. The weighted average value of segmental peak longitudinal strains was used to calculate GLS.

### 2.4 MW analysis

MW parameters were calculated using EchoPAC software (version 203, GE Vingmed Ultrasound). The methodology of MW measurement derived from noninvasive pressure‐strain loops (PSLs) has been described previously.^8^ The peak systolic LV pressure was estimated by peak arterial pressure using a sphygmomanometer during TTE. The adjusted noninvasive LV pressure curve was demonstrated by the MW software based on the duration of the isovolumic and ejection phases. Based on GLS data obtained by STE mentioned above, MW was then quantified by calculating the rate of segmental shortening by differentiation of the strain curve and multiplying this value by the instantaneous LV pressure.

**Figure 2:**
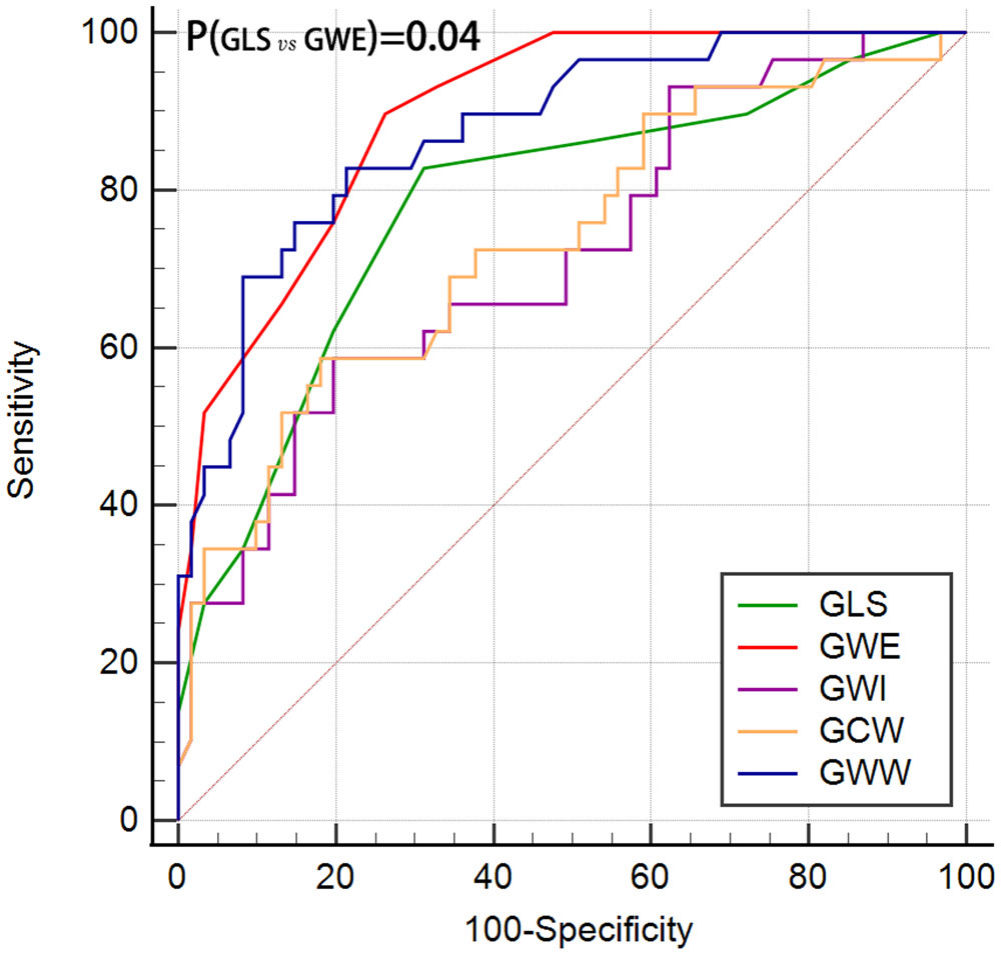
Receiver operating characteristic (ROC) curves showing the diagnostic value of global longitudinal strain (GLS) and global myocardial work (GMW) parameters for the detection of nonregional wall motion abnormality (RWMA) coronary atherosclerotic diseases (CAD) patients with high‐Gensini score (GS). Comparison of the ROC curves of GLS and global myocardial work efficiency (GWE), global myocardial work index (GWI), global constructive work (GCW), global wasted work (GWW). GWE was superior to GLS with an area under ROC curves (AUC) of 0.897 (*p* = .04).

The following indices were calculated automatically by the software: global myocardial work index (GWI), total work within the area of the LV PSL calculated from mitral valve closure to mitral valve opening; global constructive work (GCW), global MW performed during LV shortening in systole and LV lengthening during the isovolumic relaxation phase; global myocardial wasted work (GWW), global myocardial work performed during LV lengthening in systole and LV shortening during the isovolumic relaxation phase; global myocardial work efficiency (GWE), the ratio between GCW and the sum of GCW and GWW. Supporting Information S1: Figure 2 represents a typical diagram of GMW parameters from one patient (Supporting Information S1: Figure 2A–C) with no angiographic evidence of CAD (GS = 0) and one patient (Supporting Information S1: Figure 2D–F) with severe CAD (GS = 42).

### 2.5 CAG examination and gensini scoring system

All coronary angiographic images were analyzed by two interventional cardiologists who were blinded to the clinical and the echocardiographic results of the patients. The Gensini scoring system was used to evaluate the severity of coronary artery stenosis.^2^ In brief, the calculation of GS was initiated by assigning a severity score to each coronary stenosis. Subsequently, each lesion score was multiplied by a coefficient that takes into account the importance of the lesion's position in coronary circulation. (Detailed in Supporting Information S1: Table 1). In the case of a disagreement, a third interventional cardiologist who was unaware of the laboratory results and the nature of the study evaluated the coronary angiograms and determined the GS. Patients with GSs greater than 0 were defined as having CAD. No CAD was defined as patients without significant coronary stenosis (GS = 0).

In present study, the tertile stratification was used to divided CAG‐confirmed CAD patients into the low‐GS, middle‐GS, and high‐GS groups, with GSs of ≤16, 17–38, >38, respectively.^16^, ^17^

### 2.6 Variability analysis

To assess the reliability of the measurements of GLS, GWI, GCW, GWW, and GWE, a subset of 15 randomly selected patient sets underwent repeated measurements. The observer, who was unaware of the previous analysis results, measured these parameters twice with a 1‐week interval to evaluate intra‐observer variability. Interobserver variability was assessed between two independent observers, both blind to each other's results and patients' clinical data. Intra‐ and interobserver variabilities were calculated using the intra‐class correlation coefficient (ICC).

### 2.7 Statistical analysis

All statistical analyses were performed on SPSS version 25.0 and MedCalc (Version 20.0). Normally distributed continuous variables are presented as mean ± standard deviation (SD), while nonnormally distributed continuous variables are presented as median (25th and 75th percentiles). The normal distribution of continuous variables was tested using the Shapiro‐Wilk test. Categorical variables are presented as numbers and percentages. Intergroup comparisons of normally distributed continuous variables were conducted using the independent student's *t*‐test and one‐way analysis of variance (ANOVA) test. The Mann–Whitney *U* test was used for nonnormally distributed continuous variables. The chi‐square test was used for categorical variables. Receiver operating characteristic (ROC) analysis was performed to determine the optimal cut‐off values of GLS and MW parameters for predicting high‐GS. A two‐tailed *p*‐value < .05 was considered statistically significant.

## 3 RESULTS

### 3.1 Patient characteristics

Based on the CAG, all individuals (mean age, 61.2 ± 11.0 years; 70 male) were categorized into two groups: CAD patients (*n* = 90) and non‐CAD patients (defined as the control group, *n* = 34). Among the CAD patients, three subgroups were identified based on the tertiles of GS: low‐GS group (*n* = 30, 33.3%), mid‐GS group (*n* = 31, 34.4%), and high‐GS group (*n* = 29, 32.2%).

Table 1 presents the baseline clinical variables of the patients. Hyperlipidemia was more prevalent in the CAD group, particularly in the high‐GS group, while no significant differences were observed for other variables across the CAD groups. Conventional echocardiographic data are shown in Supporting Information S1: Table 2. The interventricular septal thickness during diastole (IVSd) was increased in the high‐GS group compared to the control group, while no significant differences were found in other conventional echocardiographic parameters. Notably, LVEF showed no significant difference between the CAD and control groups.

**Table 1:** Baseline characteristics of the study population.

Variable	No CAD (*n* = 34)	CAD patients (*n* = 90)	Low‐GS CAD (*n* = 30)	Mid‐GS CAD (*n* = 31)	High‐GS CAD (*n* = 29) (*n* = 19)
Age (years)	58.3 ± 11.3	62.3 ± 10.8	63.6 ± 11.0	61.8 ± 11.7	61.4 ± 9.6
Male sex, *n* (%)	17 (50.0%)	53 (58.9%)	17 (56.7%)	18 (58.1%)	18 (62.1%)
Heart rate (bpm)	70.0 (63.8, 77.8)	69.0 (62.0, 75.3)	67.5 (62.0, 75.3)	69.0 (62.0, 75.0)	70.0 (64.0, 77.5)
Body mass index (kg/m^2^)	23.5 (20.7, 25.2)	24.2 (22.0, 25.6)	24.3 (22.3, 25.7)	23.6 (21.0, 25.1)	24.7 (23.7, 25.9)
SBP (mmHg)	127.2 ± 12.5	130.6 ± 14.7	128.5 ± 13.5	128.8 ± 15.7	132.4 ± 13.2
DBP (mmHg)	77.9 ± 9.3	80.0 ± 9.9	79.7 ± 9.0	81.9 ± 9.1	77.0 ± 10.5
Hyperlipidemia, *n* (%)	3 (8.8%)	23 (25.6%)*	5 (16.7%)	7 (22.6%)	11 (37.9%)*
Hypertension, *n* (%)	10 (29.4%)	33 (36.7%)	11 (36.7%)	8 (25.8%)	14 (48.3%)
Diabetes mellitus, *n* (%)	2 (5.9%)	10 (11.1%)	3 (10.0%)	4 (12.9%)	3 (10.3%)
Current smokers, *n* (%)	10 (29.4%)	32 (35.6%)	10 (33.3%)	10 (32.3%)	12 (41.4%)
Family history of CAD	6 (17.6%)	15 (16.7%)	4 (13.3%)	7 (22.6%)	4 (13.8%)
Cardiac rhythm					
Sinus rhythm	33 (97.1%)	86 (95.6%)	30 (100%)	29 (93.5%)	27 (93.1%)
First‐degree AV block	1 (2%)	4 (4%)	0 (0%)	2 (6.5%)	2 (6.9%)
Gensini score	N/A	28.0 (12.0, 58.0)	9.5 (5.8,12.0)	28.0 (20.0, 32.0)	76.0 (60.0, 92.0)
Medications					
β‐blockers	6 (17.6%)	15 (16.7%)	4 (13.3%)	5 (16.1%)	6 (20.7%)
Statins	9 (26.5%)	30 (33.3%)	5 (16.7%)	7 (22.6%)	6 (20.7%)
ARBs	8 (23.5%)	20 (22.2%)	6 (20.0%)	5 (16.1%)	9 (31.0%)
ACE inhibitors	7 (20.6%)	24 (26.7%)	7 (23.3%)	8 (25.8%)	9 (31.0%)
Calcium channel blockers	9 (26.5%)	28 (31.1%)	9 (30.0%)	9 (29.0%)	10 (34.5%)

*Note*: Data are expressed as mean ± SD, median (25th and 75th percentiles) or as number (percentage).

Abbreviations: ACE, angiotensin‐converting‐enzyme; ARB, angiotensin II receptor blocker; CAD, coronary artery disease; DBP, diastolic blood pressure; SBP, systolic blood pressure.

* Significantly different (*p* < .05) compared with the control group.

### 3.2 GLS and GMW parameters in CAD groups and control subjects

GLS and GMW variables in all participants were summarized in Table 2. Compared to control subjects, GLS, GWE, GWI, and GCW in CAD group were significantly decreased, while GWW was increased in CAD patients (*p* < .05).

**Table 2:** GLS and GMW parameters in the study population.

Variable	No CAD (*n* = 34)	CAD patients (*n* = 90)	Low‐GS CAD (*n* = 30)	Mid‐GS CAD (*n* = 31)	High‐GS CAD (*n* = 29) (*n* = 19)
GLS (%)	19.5 (17.8, 21.2)	18.0 (16.0, 19.0)*	18.5 (17.0, 21.0)	18.0 (16.0, 19.0)*	16.0 (14.0, 17.0)*
GWE (%)	96.0 (95.0, 97.0)	93.0 (90.0, 95.0)*	95.0 (93.0, 98.0)	94.0 (92.0, 95.0)*	89.0 (87.5, 91.0)*
GWI (mmHg %)	1984.1 ± 303.8	1802.5 ± 381.8*	1992.5 ± 395.9	1838.7 ± 291.6	1567.3 ± 335.4*
GCW (mmHg %)	2255.1 ± 292.2	2081.8 ± 355.6*	2247.3 ± 334.2	2151.3 ± 274.7	1836.3 ± 329.0*
GWW (mmHg %)	92.0 (60.2, 120.0)	124.0 (97.3, 182.2)*	86.0 (47.2, 137.5)	112.0 (100.0, 145.0)*	184.0 (144.5, 251.0)*

*Note*: Data are expressed as mean ± SD or median (25th and 75th percentiles).

Abbreviations: GCW, global constructive work; GLS, global longitudinal strain; GMW, global myocardial work; GS, gensini score; GWE, global myocardial work efficiency; GWI, global myocardial work index; GWW, global wasted work.

* Significantly different (*p* < .05) compared with the control group.

In the CAD, no significant differences were observed in GLS and all GMW parameters in the low‐GS group. GLS and GWE were decreased, while GWW was increased in mid‐GS group. In high‐GS group, GLS, GWE, GWI, and GCW were significantly decreased and GWW was significantly increased.

### 3.3 GLS & GMW variables in low‐, mid‐, and high‐GS groups

GLS, which is a conventional parameter for evaluating myocardial strain, exhibited a decrease in the high‐GS group (Figure 1A). Our MW analysis revealed lower GWE, GWI, and GCW in the high‐GS group compared to the other two groups, illustrating the relationship between LV MW and the severity of coronary artery stenosis (Figure 1B–D). Consistent with these findings, GWW, which represents global wasted MW, was increased in the high‐GS group (Figure 1E).

Additionally, we observed an increase in GWW in mid‐GS group compared with low‐GS group, while no significant differences were found in GLS, GWE, GWI, and GCW.

### 3.4 The area under ROC curves (AUC)

AUC values for GLS, GWE, GWI, GCW, and GWW for identifying severe coronary lesions (high‐GS) were 0.781, 0.897, 0.717, 0.735, and 0.875, respectively (Table 3). Among these parameters, GWE exhibited superior discriminatory ability compared to GLS and other GMW parameters, with an AUC of 0.897 (Figure 2). The optimal cutoff value of GWE for assessing non‐RWMA in CAD patients with severe coronary lesions (high‐GS) was 92.0%, with a sensitivity of 89.7% and a specificity of 73.8% (Table 3). The positive predictive value and negative predictive value of GWE for predicting high‐GS were 61.9% and 93.8%, respectively.

**Table 3:** Receiver operating characteristic curve analysis for the detection of high‐GS CAD.

	GLS (%)	GWE (%)	GWI (mmHg%)	GCW (mmHg%)	GWW (mmHg%)
AUC	0.781	0.897	0.717	0.735	0.875
AUC 95% CI	0.682–0.862	0.815–0.951	0.613–0.807	0.631–0.822	0.788–0.935
Cutoff value	≤17	≤92	≤1620	≤1912	>142
Sensitivity	82.8%	89.7%	58.6%	58.6%	82.8%
Specificity	68.9%	73.8%	80.3%	82.0%	78.7%
Positive predictive values	55.8%	61.9%	58.6%	60.7%	64.9%
Negative predictive values	89.4%	93.8%	80.3%	80.6%	90.6%

Abbreviations: AUC, area under the curve; GCW, global constructive work; GLS, global longitudinal strain; GWE, global myocardial work efficiency; GWI, global myocardial work index; GWW, global wasted work.

### 3.5 Inter‐ and intra‐observer variability

The inter‐ and intra‐observer variability for GLS and GMW parameters is summarized in Supporting Information S1: Table 3. All parameters demonstrated good repeatability, with ICC values > 0.80.

## 4 DISCUSSION

Myocardial deformation analysis based on speckle tracking imaging has become a reliable and comprehensive assessment of myocardial characteristics and systolic LV function.^6^ Previous studies have reported a correlation between myocardial mechanical strain and the severity of coronary artery stenosis in EF‐preserved CAD patients.^7^, ^18^ However, it is important to note that STE analysis is limited in its dependence on the load. An increase in afterload has been shown to negative correlation with strain, potentially leading to misunderstanding about myocardial contractility.^10^, ^19^ In contrast, MW analysis has demonstrated superior value in evaluating LV contractility compared to other echocardiographic parameters, including LVEF and strain.^8^, ^9^, ^10^ Notably, a previous study has shown a strong correlation between MW and regional myocardial glucose metabolism as assessed by F‐fluorodeoxyglucose positron emission tomography (18F‐FDG PET) evaluation.^8^ By estimating myocardial energetics, MW methods provide valuable insights as the PSL area reflects myocardial oxygen consumption and metabolism.

Our present study demonstrated that noninvasive MW analysis derived from PSLs provides quantifiable information for identifying CAD patients, even in the absence of visually detectable RWMAs. All GMW parameters in the CAD group exhibited significant changes, while LVEF showed no significant difference (63.9 ± 3.1 vs. 62.7 ± 3.6, *p* = .12). These findings suggest that MW performance is impaired in these patients, despite the LV ejection function appearing unchanged.

In a recent study, Wang et al. investigated the relationship between LV global myocardial work (GMW) and the degree of coronary artery stenosis in suspected CAD patients.^20^ The patient cohort was divided into four groups based on the stenosis rate of the main coronary vessels. Additionally, Zhang et al. explored the value of global and regional MW parameters in predicting high‐risk stable coronary artery disease (SCAD) patients with normal wall motion and preserved LV function. High‐risk SCAD was defined as left main coronary artery diameter stenosis ≥50%, 3‐vessel disease (diameter stenosis ≥70%), or 2‐vessel disease involving the proximal LAD.^21^

In our present study, CAD severity was assessed using the Gensini scoring system. Unlike conventional assessments that focus on the main vessels or the most severe luminal stenosis, the Gensini scoring system provides a more comprehensive evaluation by considering the degree of multivessel luminal narrowing and anatomical location, thereby offering valuable information.^2^, ^3^ Our findings indicate that GLS, GWE, GWI, and GCW were significantly decreased in the high‐GS group, while GWW increased compared to the low‐ and mid‐GS groups. In a normal heart, different myocardial segments contract in a highly synchronized and coordinated manner.^22^ Constructive work refers to the positive work performed during LV shortening in systole and LV lengthening during the relaxation phase. In contrast, wasted work is defined as the work generated during systolic lengthening and diastolic shortening in dyssynchronous contractions.^10^ Therefore, in patients with high‐GS, severe myocardial ischemia may lead to reduced myocardial oxygen and blood flow, resulting in decreased MW performance and increased wasted work.

Furthermore, the ROC results demonstrated that GWE was the most powerful predictor of identifying high‐GS in patients with CAD with an AUC value of 0.897. It was found to be superior to GLS (AUC value = 0.781, *p* = .04). A GWE value below 92.0% allowed a sensitivity of 89.7% and a specificity of 73.8% in evaluating non‐RWMA CAD patients with severe coronary lesions. Global myocardial work efficiency (GWE) is defined as followed: GWE = constructive work/(wasted work + constructive work), which tend to be a more objective indicator in different patients regardless of individual variation.^9^ Similarly, Wang's study also revealed that GWE was the most significant predictor among all MW parameters for detecting severe stenosis.^20^ Inconsistent with our results, they found that all GMW parameters changed in moderate stenosis group compared to mild group. While in our study, we noted an increase in GWW in mid‐GS group compared with low‐GS group, while no significant differences were found in other parameters. Moreover, Wang et al revealed that there were significant differences in all global MW parameters except GWW between moderate and severe stenosis group.^20^ Present study demonstrated all GMW parameters changed significantly in high‐GS group. Our results suggested that in our studying population, with the aggravation of coronary stenosis, The changes of GWW may be more sensitive and earlier to myocardial ischemia.

In our previous study on the change in left ventricular dyssynchrony (LVD) index in non‐RWMA CAD patients, we found that CAD patients tended to show increased LVD compared to the negative groups.^23^ Florian Schrub et al. reported that the MW may play an important role in detecting the presence of LVD in dilated cardiomyopathy patients.^24^ Importantly, significant change in GWW and GWE, as well as a heterogeneous distribution of MW showed observably association with LVD using echocardiography. In fact, other studies have shown that a delayed onset of segmental subendocardial thinning occurs at an early stage of ischemic hypoperfusion, which can induce regional asynchronous contraction, leading to presence of LVD.^25^, ^26^ These alterations may be associate and interact with the heterogeneous distribution of MW, resulting in an impairment of global MW efficiency and overall myocardial performance.^27^ Whether MW and LVD interact with each other or are just present as two different symptoms caused by coronary ischemia deserves further investigation.

Moreover, regional myocardial strain and work analysis may be a promising method for evaluating segmental performance in the perfusion area of coronary artery lesions. Pieter et al. reported that in patient with ST‐segment elevation myocardial infarction, RWE and RWI values were decreased in the anterior wall, with compensatory increases in the lateral wall.^28^ Boe E et al. revealed that the presence of a region of reduced MWI in patients with NSTE‐ACS identified patients with ACO.^9^ These findings highlight the potential of regional MW parameters in diagnosing cardiovascular disease. The present study aimed to investigate global MW in CAD patients without RWMA. Further study will focus on the relationship between regional MW analysis and ischemic coronary perfusion areas.

This study has limitations inherent to its retrospective nature and the study population. The study recruited a limited number of participants from a single center.

## 5 CONCLUSION

LV PSLs can be applied to estimate MW in CAD patients, and WM parameters assessment provides incremental value in detecting CAD in non‐RWMA patients. The proposed GWE, as a representative parameter of MW, showed the optimal performance in predicting high‐GS which could be considered a potential predictive factor for detecting severe CAD in CAD patients without RWMA.

## AUTHOR CONTRIBUTIONS

Conception, design, or critical revision of the manuscript was provided by Pingyang Zhang. Acquisition, analysis, or interpretation of data were supplied by Miao Li and Yuhao Wang. Drafting of the manuscript was done by Miao Li. Statistical analysis was contributed by Lin Li. Statistical analysis in the modification process was contributed by Rong Qian. Administrative, technical, or material support was provided by Wenfang Wu.

## CONFLICT OF INTEREST STATEMENT

The authors declare no conflict of interest.

## Supporting information

Supporting information.

## Data Availability

All data generated or analyzed during this study are included in this article. Further enquiries can be directed to the corresponding author.
