# Supplementary material for: Global myocardial work in coronary artery disease patients without regional wall motion abnormality: Correlation with Gensini‐score
Source: Clin Cardiol. 2023 Nov 28;47(2):e24193. doi: 10.1002/clc.24193 (PMC10823439; doi:10.1002/clc.24193)
Supplement: Supplementary file 1 — Supporting information. [file CLC-47-e24193-s001.docx]

**Global myocardial work in coronary artery disease patients without regional wall motion abnormality: correlation with Gensini-score**

Supplementary Figure 1

Supplementary Figure 2

Supplementary Table 1

Supplementary Table 2

Supplementary Table 3


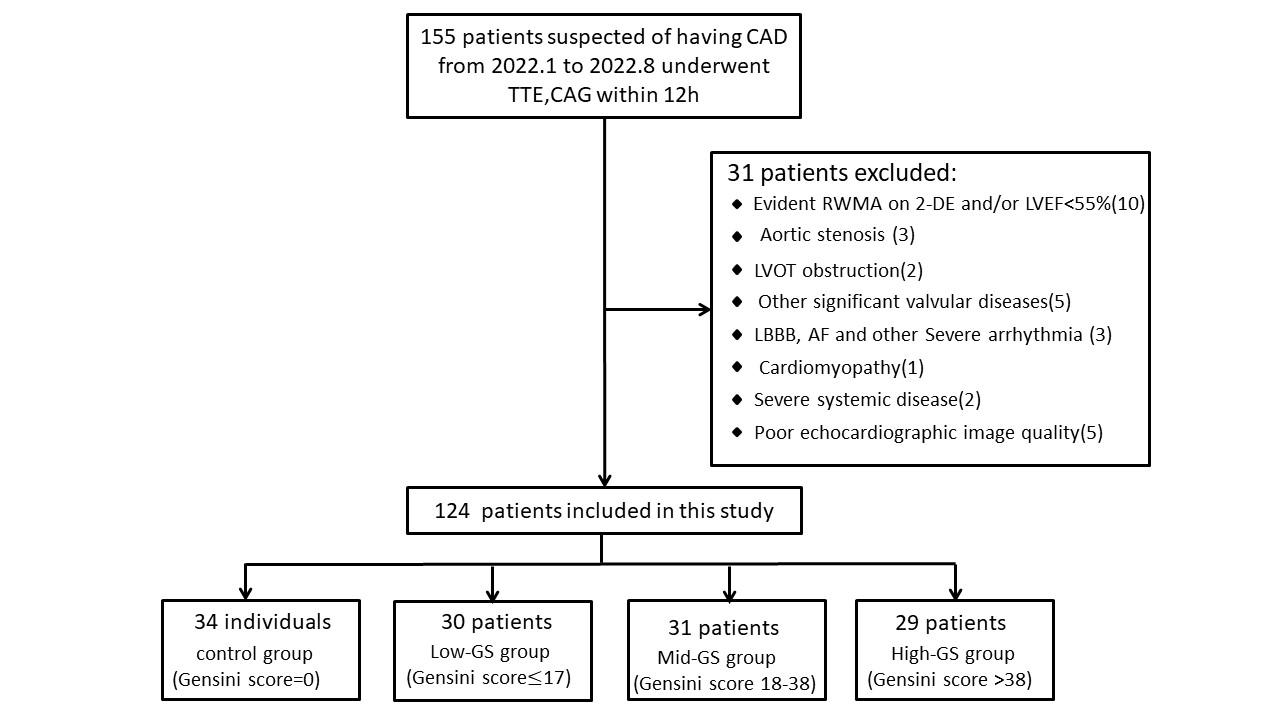


**Supplementary.Fig.1 Inclusion of patients in the study.**


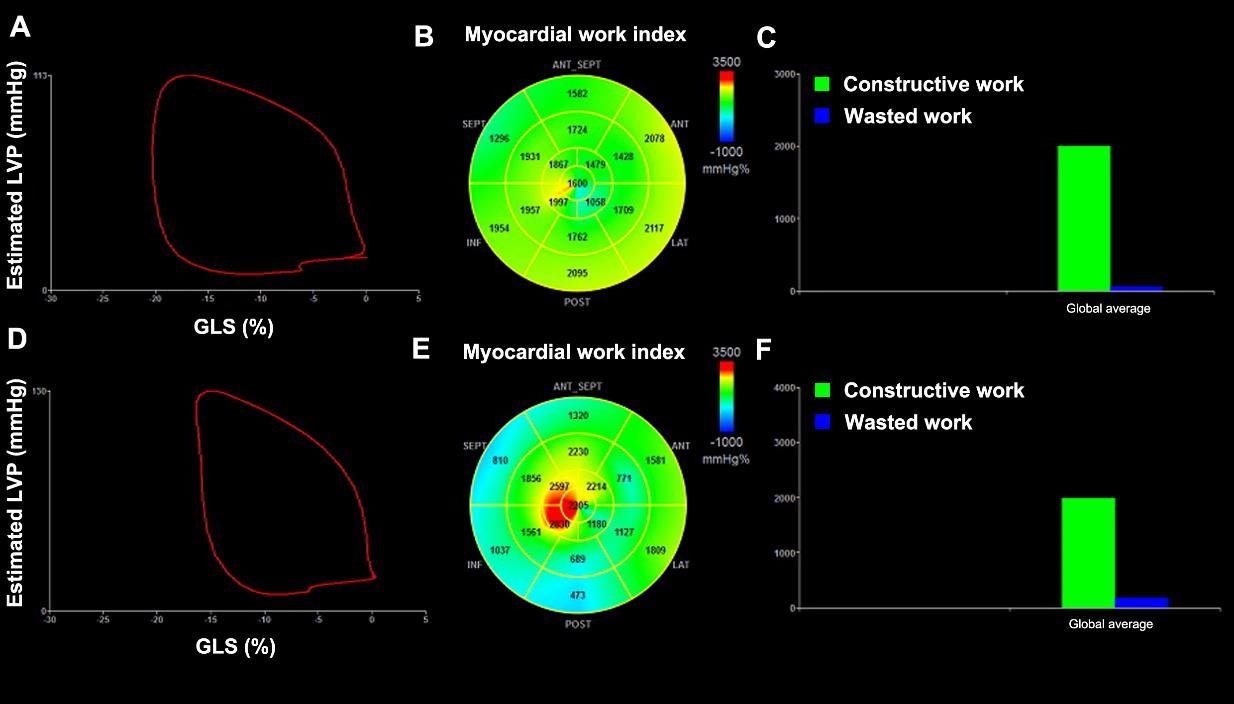


**Supplementary.Fig.2 Representational global myocardial work parameters diagram.** Non-invasive left ventricular pressure-strain loop from one patient with no angiographic

evidence of CAD (A) and one patient with severe CAD(GS=42) (D) respectively. The area

within the loop is estimated as global MW. Visually the LV pressure-strain loop in fig. D has a

smaller area. Segmental bull’s-eye MW plot from one patient with no angiographic evidence

of CAD (B) and one patient with severe CAD (E.) respectively. On the bull’s-eye plot, there is

significant reduction of myocardial work index in fig. E which demonstrated as blue-coded

areas. GCW and GWW diagram from one patient with no angiographic evidence of CAD (C.)

and one patient with severe CAD (F.) respectively. GS, Gensini score; MW, myocardial work;

GCW, global constructive work; GWW, global wasted work.

|  | **Supplementary Table 1. Gensini scoring system** | |  |
| --- | --- | --- | --- |
| **coronary stenosis** | **severity score** | **lesion’s position** | **Position score** |
| 1%-24% | 1 | LM | 5 |
| 25%-49% | 2 | Proximal segment of the LAD or LCX | 2.5 |
| 50%-74% | 4 | Mid segment of the LAD | 1.5 |
| 75%-90% | 8 | Apical segment of the LAD and first diagonal branch | 1 |
| 91%-99% | 16 | Mid and apical segment of LCX | 1 |
| Total occlusion | 32 | RCA | 1 |
|  |  | Other subbranch | 0.5 |

LM, left main coronary artery; LAD, left anterior descending; LCX, left circumflex artery; RCA, right coronary artery.

Calculation of the Gensini score was initiated by giving a severity score to each coronary stenosis. Each lesion score was multiplied by a coefficient that considers the importance of the lesion’s position in coronary circulation.

**Supplementary Table 2. Conventional echocardiographic parameters of the study population**

| **Variable** | **No CAD (*n*=34)** | **CAD patients (*n*=90)** | **Low-GS CAD(*n*=30)** | **Mid-GS CAD(*n*=31)** | **High-GS CAD(*n*=29)** |
| --- | --- | --- | --- | --- | --- |
| LVDd (mm) | 47.6±4.7 | 47.1±3.8 | 47.6±3.6 | 47.1±4.1 | 46.5±3.8 |
| LVDs (mm) | 30.9±3.6 | 30.3±2.8 | 30.8±2.6 | 30.3±3.2 | 29.7±2.5 |
| IVSd (mm) | 9.0(9.0,10.0) | 10.0(9.0,10.3) | 10.0(9.0,10.0) | 10.0(9.0,10.0) | 10.0(9.0,11.0) * |
| LVPWd (mm) | 9.0(8.8,9.0) | 9.0(9.0,10.0) | 9.0(9.0,10.0) | 9.0(9.0,9.0) | 9.0(9.0,10.0) |
| Biplane LVEF (%) | 65.0(60.8,66.0) | 64.0(60.0,66.0) | 64.0(60.8,65.0) | 64.0(60.0,66.0) | 63.0(60.0,66.0) |
| SV / BSA (ml/ m^2^) | 39.8(34.7,45.7) | 37.4(33.5,42.8) | 37.8(33.4,43.6) | 37.9(34.3,42.7) | 36.0(30.8,43.0) |
| E/A | 0.8(0.70,1.16) | 0.8(0.7,1.1) | 0.9(0.7,1.2) | 0.8(0.7,1.1) | 0.8(0.7,1.1) |
| E/e | 10.5(7.6,13.5) | 10.5(8.0,13.2) | 10.5(8.3,13.7) | 9.9(7.6,12.3) | 10.5(7.8,13.7) |

CAD, coronary artery disease; LVDd, LV dimension in end diastole; LVDs, LV dimension in end systole; IVSd, interventricular septal thickness in diastole; LVPWd, LV posterior wall thickness in diastole; LVEF, left ventricular ejection fraction; SV, stroke volume; BSA, body surface area.

Data are expressed as mean ± SD or median (25th and 75th percentiles).

*Significantly different (P <0.05) compared with the control group.

**Supplementary Table 3.**

**ICCs for intra- and interobserver variability for GLS and GMW parameters**

| Variable | Inter-observer variability |  | Intra-observer variability | |
| --- | --- | --- | --- | --- |
|  | ICC | 95%CI | ICC | 95%CI |
| GLS | 0.911 | 0.757-0.969 | 0.855 | 0.622-0.949 |
| GWE | 0.943 | 0.839-0.981 | 0.902 | 0.734-0.966 |
| GWI | 0.908 | 0.748-0.968 | 0.812 | 0.528-0.933 |
| GCW | 0.912 | 0.759-0.970 | 0.906 | 0.744-0.967 |
| GWW | 0.933 | 0.814-0.977 | 0.898 | 0.724-0.965 |

ICC, intra-class correlation coefficient; CI, confidence interval; GMW, global myocardial work; GLS, global longitudinal strain; GWE, global myocardial work efficiency; GWI, global myocardial work index; GCW, global constructive work; GWW, global wasted work.
